# Longitudinal changes in COVID-19 clinical measures and correlation with the extent of CT lung abnormalities

**DOI:** 10.7150/ijms.51279

**Published:** 2021-01-16

**Authors:** Min Zhou, Chengjun Dong, Chungao Li, Yuhui Wang, Huijun Liao, Heshui Shi, Alexander Peter Lin, Jing Wang, Yue Hu, Chuansheng Zheng

**Affiliations:** 1 Department of Radiology, Union Hospital, Tongji Medical College, Huazhong University of Science and Technology, Wuhan, Hubei, China; 2 Hubei Province Key Laboratory of Molecular Imaging, Wuhan, Hubei, China; 3 Cancer Center, Union Hospital, Tongji Medical College, Huazhong University of Science and Technology, Wuhan, Hubei, China; 4 Center for Clinical Spectroscopy, Brigham and Women's Hospital, Harvard Medical School, Boston, MA, United States

**Keywords:** corona virus disease 2019, severe acute respiratory syndrome coronavirus 2, CT, laboratory parameters, longitudinal changes

## Abstract

**Rationale:** To assess the longitudinal changes and relationships of clinical measures and extent of CT lung abnormalities in COVID-19.

**Methods:** 81 patients with COVID-19 were prospectively enrolled and followed until discharge. CT scores were quantified on a basis of a CT scoring system where each lung was divided into 3 zones: upper (above the carina), middle, and lower (below the inferior pulmonary vein) zones; each zone was evaluated for percentage of lung involvement on a scale of 0-4 (0, 0%; 1, 0-24%; 2, 25% - 49%; 3, 50% -74%; 4, >74%).Temporal trends of CT scores and the laboratory parameters characteristic of COVID-19 were analyzed. Correlations between the two were determined at three milestones (initial presentation, worst CT manifestation, and recovery finding before discharge). Their correlations with duration to worst CT manifestation and discharge from symptom onset were evaluated.

**Results:** CT scores peaked during illness days 6-11 (median: 5), and stayed steady. C-reactive protein and lactate dehydrogenase increased, peaked on illness days 6-8 and 8-11 (mean: 23.5 mg/L, 259.9 U/L), and gradually declined. Continual decrease and increase were observed in hemoglobin and lymphocyte count, respectively. Albumin reduced and remained at low levels with a nadir on illness days 12-15 (36.6 g/L). Both initial (r = 0.58, 0.64, p < 0.05) and worst CT scores (r = 0.47, 0.65, p < 0.05) were correlated with C-reactive protein and lactate dehydrogenase; and CT scores before discharge, only with albumin (r = -0.41, p < 0.05). Duration to worst CT manifestation was associated with initial and worst CT scores (r = 0.33, 0.29, p < 0.05). No parameters were related to timespan to discharge.

**Conclusion:** Our results illustrated the temporal changes of characteristic clinical measures and extent of CT lung abnormalities in COVID-19. CT scores correlated with some important laboratory parameters, and might serve as prognostic factors.

## Introduction

The pandemic of corona virus disease 2019 (COVID-19), caused by severe acute respiratory syndrome coronavirus 2 (SARS-CoV-2), has lasted for more than 1 year, and poses a major threat to global health 1. As of Jan. 10, 2021 88,387,352 confirmed cases and 1,919,204 deaths are reported, and the numbers keep increasing sharply 2.

The CT manifestations and characteristic laboratory abnormalities in COVID-19 pneumonia have been reported. The most commonly seen CT findings in COVID-19 pneumonia are bilateral ground-glass opacity with subpleural distribution 3, 4. In addition, prior studies have reported that COVID-19 was characterized by some laboratory abnormalities, including but not limited to lymphocytopenia and elevated levels of C-reactive protein (CRP) and lactate dehydrogenase (LDH) 5-8. However, the natural history of this highly contagious disease remains poorly understood. A recent article described the longitudinal course of CT manifestations and some clinical measures, concluding that the progression course of CT pattern was later than the clinical parameters within the first two weeks, and those two improved synchronously in the 4th week 9. Nevertheless, the study sample only includes 17 patients, and thus the temporal trends and relationships of the extent of CT lung abnormalities and the clinical measures need to be validated in a larger sample size. Also, the correlations between the severity of CT lung abnormalities and the laboratory parameters over the course of the disease, and the prognostic role of those two warrant further investigation. A better understanding of these questions will not only provide insight into the clinical course of COVID-19, but also facilitate better patient management in clinical practice. We therefore performed this study to compare the temporal trends of the extent of CT lung abnormalities and the laboratory parameters in a relatively larger group of 81 patients with COVID-19, to determine the association between the two across the course of COVID-19 pneumonia, and their correlation with illness duration.

## Materials and methods

The Institutional Review Board of Union Hospital, Tongji Medical College, Huazhong University of Science and Technology approved this prospective observational study whereby written informed consent was waived due to the urgent need to collect date for this highly infectious epidemic crisis.

### Patients

Patient enrollment occurred between January 16, 2020 and February 17, 2020. All patients who were admitted to the isolation wards of Union Hospital with suspected COVID-19 pneumonia were screened. The inclusion criteria were: (1) The patient had confirmed diagnosis of COVID based on positive real-time reverse transcriptase-polymerase chain reaction (rRT-PCR) tests for SARS-CoV-2 from oropharyngeal swabs; (2) The patient also had to have at least 1 CT scan showing lung abnormalities before or after admission; (3) the electronic records for the patient had to be available. Patients were followed until they were discharged from hospital, until death, or until the end of the study (June 30, 2020) if they were still in the hospital. Patients who required ICU admission or died, who were transferred to other hospitals during treatment, or who were still in the hospital at the end of the study were excluded.

This prospective patient cohort had been reported in a prior study that discussed the temporal changes of CT findings 3. However, in this study, only patients that completed successful treatment without ICU admission were evaluated.

### Treatment and Discharge Criteria

Treatment and discharge criteria for patients with COVID-19 pneumonia were in accordance to the Guidelines for the Diagnosis and Treatment of COVID-19 Pneumonia published by the National Health Commission of the People's Republic of China 10. Patients were discharged if they: (1) were afebrile for at least 72 hours; (2) showed significant improvement of respiratory symptoms; (3) showed evidence of improvement on chest CT or radiograph; and (4) had two consecutive negative SARS-CoV-2 rRT-PCR results at least 24 hours apart.

### CT Examination

The chest CT scans were obtained on either Discovery 750HD (GE Medical Systems, Milwaukee, Wis) or TOSHIBA Activion 16 (Toshiba, Tokyo, Japans) by using 120 kVp and adaptive tube current, with breath-holds at end inspiration. The images were reconstructed with 1.25 mm section thickness and interval of 1.25 mm. 5.0 mm section thickness and 5.0 mm interval were sometimes utilized for reconstruction on TOSHIBA Activion 16 for a faster transfer of images to PACS due to the large number of patients presenting for emergency CT scans and quick review. Every CT in out department was used the standard chest CT protocol, and performed calibration every week to ensure reliable work performance and image quality.

Patients were assigned to whichever scanner that was available at the time of CT exams.

### CT Image Interpretation

Five experienced radiologists in 2 groups (group one: YHW, CGL, and XZ, with an experience of 11, 12 and 3 years in radiology, respectively; group two: CJD and QQR, with an experience of 5 and 6 years in radiology, respectively) interpreted the CT images for the extent of lung abnormalities with a method published previously 3, 11. Briefly, the extent of lung abnormalities were quantified on a basis of a CT scoring system where the lungs were divided into 6 zones and the involvement of each zone was evaluated on a scale of 0-4. The overall CT score was the summation of scores from all 6 lung zones with the maximum possible score of 24. Multiple chest CT scans obtained for a single patient were reviewed by the same group based on the consensus of the radiologists. The κ-value for interobserver agreement of CT scores between the two groups on the basis of 30 CT scans randomly selected from our patient cohort was 0.84.

### Laboratory Parameters

Laboratory parameters which were reported abnormal in more than 50% of the patient samples in prior studies were selected (Supplementary Table S1), and retrieved from the medical records. These laboratory parameters included CRP, LDH, hemoglobin, lymphocyte count, albumin and interleukin-6 (IL-6).

### Milestone Selection

Three milestones during the course of illness were determined for each patient at the time of CT review as per other SARS studies 12: the time of initial CT (initial presentation), the time at which the CT appearance was the worst (worst CT manifestation), and the time at which the last CT was obtained before discharge (recovery finding before discharge). For patients whose initial CT showed the worst appearance, the initial presentation would be regarded as the "worst CT manifestation". Laboratory parameters on the same day of the milestones, if any, were extracted for correlation analysis. If no laboratory parameters were acquired on the specific date, laboratory parameters obtained the day after the specific date were used. If such data were also unavailable, laboratory parameters obtained the day before the specific date, followed by two days after or before the specific date were used.

### Statistical Analysis

The day of initial symptom onset was defined as illness day 0. Kruskal-Wallis rank sum test and Mann-Whitney U test were used to test for difference between median values of CT scores and mean values of laboratory parameters at 3 milestones. Chi-square test was applied to compare the frequency of deranged values in different time points. The correlations between CT scores and laboratory parameters at 3 milestones, and their correlation with the time to the worst CT manifestation and discharge from symptom onset were assessed by Spearman rank correlation analysis. A p value < 0.05 were considered to be statistically significant. All analyses were performed with SPSS software (version 21, IBM Corporation, NY, USA). In addition, we performed post-hoc sample size calculations to check if the sample size was enough to detect the positive correlations with type I error rate of 0.05 and power of 0.7 using the PASS 15.0 software 13.

## Results

### Patient Population

During January 16 to February 17, 2020, 107 patients were admitted to the isolation wards. Among them, 90 patients with positive SARS-CoV-2 rRT-PCR result were included. As of June 30, 2020, 9 patients were excluded due to ICU admission or death (6 patients), or transfer to other hospitals during treatment (3 patients), resulting in a total of 81 patients who were discharged and therefore enrolled in the final study. The median hospital stay was 18 days (range: 5-43), and the median illness duration from symptom onset to hospital discharge was 25 days (range: 10-55). The demographic features are summarized in Table 1.

The total numbers of CT scan and each laboratory parameter, median numbers of each exam per patient, and the median time intervals for each exam are shown in Table 2.

### Temporal changes of CT lung quantifications and laboratory parameters

**Figure 1:**
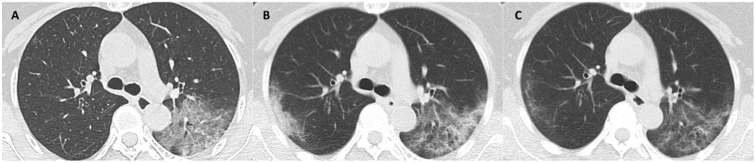
** CT scans at 3 milestones in a 65-year-old woman with COVID-19 pneumonia. (A)** Initial CT obtained on illness days 1 showed ground-glass opacity in left upper lobe. **(B)** Worst CT obtained on illness days 8 showed ground-glass opacity and consolidation with increased extent in bilateral upper lobes. **(C)** CT scan before discharge obtained on illness days 18 showed substantial residual ground-glass opacity. The patient was discharged on illness days 19.

**Figure 2:**
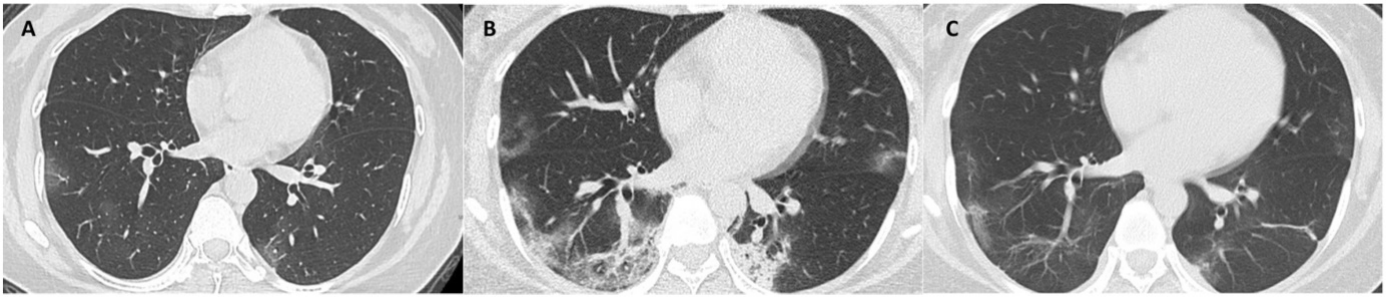
** CT scans at 3 milestones in a 33-year-old woman with COVID-19 pneumonia. (A)** Initial CT obtained on illness days 7 showed ground-glass opacity in bilateral lower lobe. **(B)** Worst CT obtained on illness days 13 showed ground-glass opacity and consolidation with largely increased extent in bilateral lower lobes. **(C)** CT scan before discharge obtained on illness days 22 showed mild residual ground-glass opacity. The patient was discharged on illness days 25.

The median days of illness since symptom onset for the first, second and third milestones were 2 (range: 0-12), 10 (1-22), and 22 (7-53), respectively. The median values of total CT scores, and the mean values of each laboratory parameter at 3 milestones are listed in Table 3. Serial CT images at 3 milestones in 2 patients are shown in Figure 1-2.

**Figure 3:**
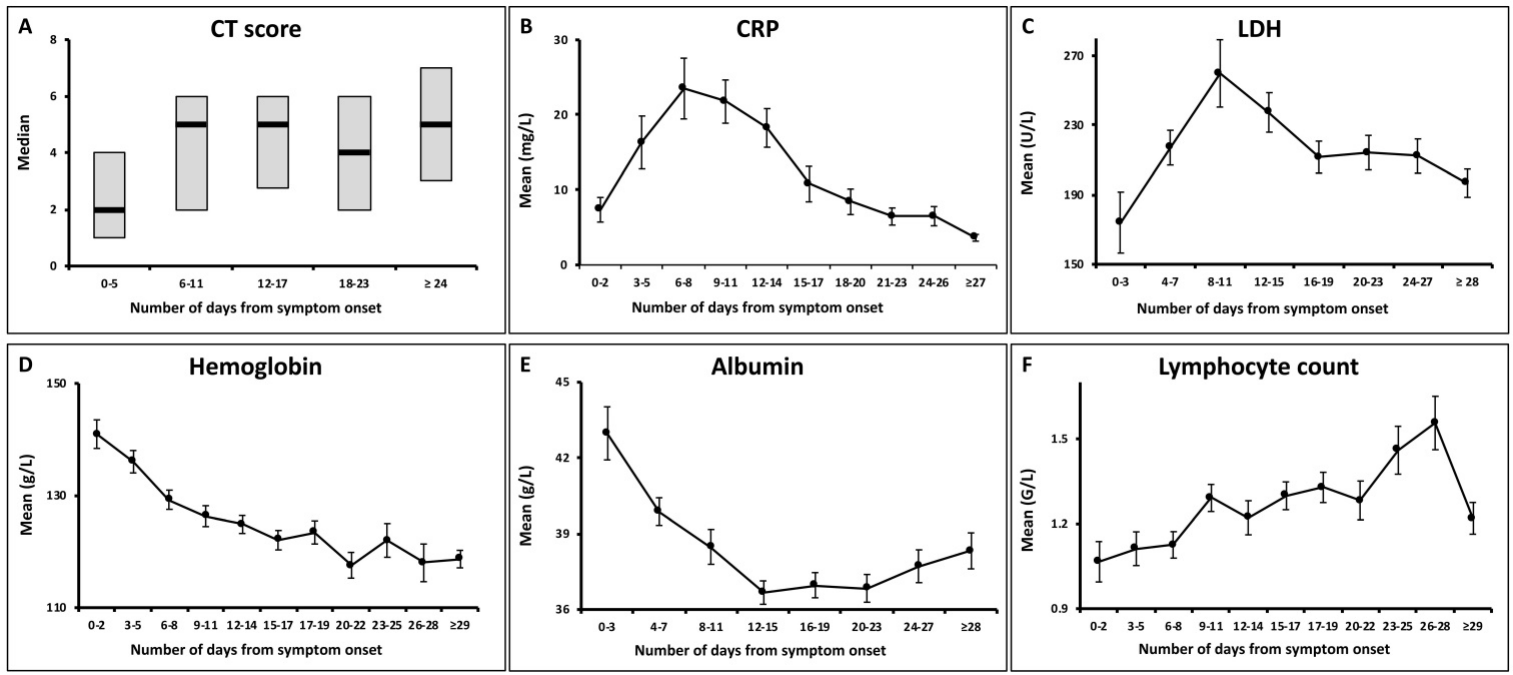
Temporal changes of total CT scores and the laboratory parameters. The point intervals on each x axis were based on the median time intervals of the corresponding exams (as shown in Table 2). CRP = C-reactive protein; LDH = lactate dehydrogenase.

There were significant changes over the 3 milestones with respect to the median values of total CT score and the mean values of CRP, lymphocyte count, hemoglobin, albumin and LDH. The trends of those parameters are shown in Figure 3.

Median CT scores showed a marked increase after symptom onset which peaked during illness days 6-11 with a median value of 5 (interquartile range: 3-7), and then persisted. No significant differences were observed between the median CT scores on illness days 6-11 and later (p = 0.62).

Both the CRP and LDH levels increased rapidly after symptom onset, and reached peak levels on illness days 6-8 and 8-11, respectively, with the mean values of 23.5 ± 29.8 mg/L and 259.9 ± 134.4 U/L (mean ± standard deviation). They gradually declined to low levels. 68/81 (84%) of patients showed normal CRP levels, and 66/75 (88%) showed normal LDH levels at the last assessment.

The hemoglobin levels showed a continual decrease during the illness course. 26/81 (32%) of patients had hypochromia at the last assessment, as compared to 5/81 (6%) of patients at initial assessment (p < 0.001).

The albumin levels reduced after symptom onset, with the nadir occurring during illness days 12-15 (mean ± SD: 36.6 ± 3.9 g/L) and persisted in low levels thereafter (p = 0.175). Hypoalbuminemia developed in 15/80 (19%) of patients at the last assessment.

A gradually increasing trend was observed for the lymphocyte count. Lymphopenia was presented in 40/81 (49%) of patients at initial assessment, compared to 12/81 (15%) of patients at the last assessment (p < 0.001).

**Figure 4:**
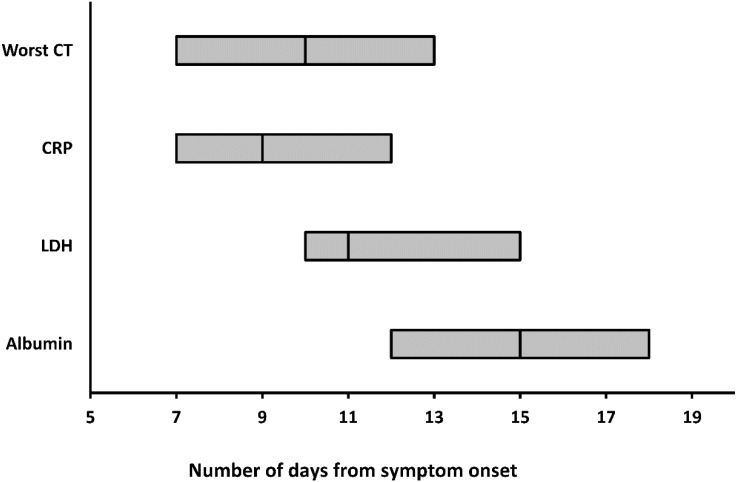
The medians and interquartile ranges of the days when worst CT, peaking CRP and LDH levels, and nadir albumin levels were reached. CRP = C-reactive protein; LDH = lactate dehydrogenase.

The worst CT, peak CRP and LDH levels, and the nadir albumin levels occurred at a median illness day 10 (interquartile range: 7-13), 9 (7-12), 11 (10-15), and 15 (12-18), respectively (Figure 4).

### Correlation between total CT scores and laboratory parameters

The correlation coefficients between total CT scores and the laboratory parameters at each milestone were listed in Table 4. At initial presentation, CT score was correlated well with the CRP and LDH levels (r = 0.58, p = 0.00; r = 0.64, p = 0.045). At worst CT manifestation, CT score was associated with both the CRP and LDH levels (r = 0.47, p =0.00; r = 0.65, p =0.00), and inversely related to the albumin levels (r = -0.43, p = 0.00). CT score was only negatively correlated with the albumin levels (r = -0.41, p = 0.00) before discharge.

### Correlation between total CT scores, laboratory parameters and the illness duration

The correlation coefficients between total CT scores, the laboratory parameters at each milestone and illness duration were shown in Table 5.

The illness duration from symptom onset to worst CT manifestation was associated with initial CT scores (r = 0.33, p = 0.01), worst CT scores (r = 0.29, p = 0.01), and negatively related to albumin (r = -0.34, p = 0.00) on worst CT manifestation day.

There was no correlation found between illness duration from symptom onset to discharge and CT scores or the laboratory parameters at each of the 3 milestones.

Of note, the illness duration from symptom onset to worst CT manifestation positively correlated with the illness duration from symptom onset to discharge (r = 0.27, p = 0.04).

### Power Analysis

The required sample sizes given type I error rate of 0.05 and power 0.7 are shown in Supplementary S2. We had enough power to detect the correlations.

## Discussion

Our results compared the progression patterns of CT lung measures and the key laboratory parameters, and showed that the severity of CT lung abnormalities correlated with some laboratory parameters, such as CRP and LDH levels on initial presentation and worst CT manifestation day. Also, our results suggested that CT scores and a few laboratory parameters mentioned above could serve as potential prognostic factors in COVID-19 pneumonia.

The CT abnormalities showed rapid deterioration since symptom onset, peaked on illness days 6-11, and persisted at high level thereafter, as similar to the prior reports 3. Both the CRP and LDH showed an approximately parallel increasing trend to that of CT scores after symptom onset, and similar peaking periods. These findings were consistent with those documented in SARS 14, 15. However, CRP and LDH gradually reduced to low levels (back to normal range in most of the patients before discharge), whereas CT scores stayed at a high level until discharge. This divergence at a later stage was different from that seen in SARS, where both radiographic and clinical measures showed improvement after worst radiograph day 12. It may indicate a longer duration of lung damage and a slower absorption rate in COVID-19 compared to SARS, which warrants further investigation. Han et al reported a trend of CT scores similar to that of our study, but a continue decrease in CRP since admission compared with the "first rising then falling" trend of CRP in our study 16. The difference in the trends of CRP was probably due to the different intervals of time axis used (every 2 days in our study vs. weeks in Han's study) and different sample sizes.

A gradually increasing trend was observed for the lymphocyte count, which was similar to that reported by Han et al 16. The hemoglobin showed a decreasing trend throughout the illness, and 26/81 (32%) of patients developed hypochromia before discharge, indicating the presence of anemia of inflammation 17. The albumin decreased and stayed in low levels, suggesting the development of liver impairment during COVID-19 18. Han et al reported an increasing trend of alanine transaminase and aspartate transaminase within the first 2 weeks in their patient cohort, indicating liver damage, which was in line with our findings 16.

Our results demonstrated that the initial CT scores and the worst CT scores were significantly correlated with both CRP and LDH levels, which is consistent with prior reports in COVID-19 and SARS 12, 19-24. CRP levels paralleled the severity of inflammation, and LDH had been proposed as an indicator of lung damage or inflammation 12, 25, 26. Those findings suggested that chest CT could be used as a valid method for the assessment of severity of infection at those time points. In contrast to us, Zhang et.al reported no correlation between CT scores of the peak and any laboratory parameters including CRP and LDH 20. This remains to be studied further in larger sample size. However, no significant correlation between CT scores and CRP or LDH levels was observed before discharge, which could be accounted for by the divergence between the trends of CT scores and CRP and LDH at later stage as mentioned above. A negative correlation was found between CT scores and albumin levels on worst manifestation day and before discharge, and albumin was the only laboratory parameter related to CT scores before discharge. It was probably because the deranged albumin values occurred mainly at the later stage of the disease.

The initial and worst CT scores were found to be associated with the illness duration as determinate by the time from symptom onset to worst CT manifestation, suggesting that the extent of CT lung abnormalities might serve as prognostic factors in COVID-19. The relationship between the extent of lung abnormalities and clinical outcome in both COVID-19 and SARS had been reported. A few studies identified CT scores on initial presentation and worst CT manifestation day as prognostic factors for death 12, 23, 27-29, 30, 31. Of note, there was no correlation found between CT scores, laboratory parameters and illness duration from symptom onset to discharge. A reasonable explanation is that patients were in variable clinical status at discharge, from complete resolution of the lung lesions to substantial lung abnormalities 3.

There are several limitations in our study. First, only discharged patients were enrolled, and the temporal changes of CT findings and laboratory parameters for patients who were admitted to ICU or died required further investigations. Second, larger sample size is needed to improve the depiction of the progression of the illness.

In conclusion, the results of this study illustrate the temporal changes of the extent of CT lung abnormalities and important laboratory parameters, and thus facilitate a better understanding of the clinical course of COVID-19. The severity of CT lung abnormalities correlates well with some key laboratory parameters, such as CRP and LDH level on initial presentation and worst CT manifestation day, and is useful for patient assessment on these milestones, but is with limited value at discharge. CT scores may serve as prognostic factors in COVID-19.

## Supplementary Material

Supplementary materials, figures and tables.

## Figures and Tables

**Table 1:** The demographic features and initial symptoms of the included patients.

Variables	Number of cases (percentage)
**Gender** (male : female)	29 : 52
**Age** (y, mean ± standard deviation)	42 ± 11
**Initial symptoms**	
Fever	47 (58%)
Cough	19 (23%)
Fatigue	20 (25%)
Pharyngalgia	6 (7%)
Rigor	7 (9%)
Headache	3 (4%)
Diarrhea	6 (7%)
Myalgia	2 (2%)
Anorexia	1 (1%)
Dizziness	1 (1%)
Dyspnea	1 (1%)

**Table 2:** The total number, median number per patient, and the median time interval of each exam.

Examsa	Total number	Median number per patient (Range)	Median time interval (Days, Range)
CT scan	346	4 (2-7)	6 (2-19)
CRP	468	5 (2-14)	3 (1-20)
LDH	266	3 (0-7)	4 (1-24)
Hemoglobin	559	7 (2-20)	3 (0-15)
Albumin	356	4 (1-14)	4 (0-24)
Lymphocyte	559	7 (2-20)	3 (0-15)
IL-6	135	1 (0-5)	7 (1-32)

^a^ CRP = C-reactive protein; LDH = lactate dehydrogenase; IL-6 = interleukin-6.

**Table 3:** The median values of total CT scores, and the mean values of each laboratory parameter at 3 Milestones.a

Parameters^b^	Initial presentation	Worst CT manifestation	Recovery finding before discharge	p value
Number of CT scans	66	81	80	-
Total CT scores	1 (1, 4)	5 (3, 7)	4 (2, 6)	0.000
CRP (mg/L)	11.3 ± 16.3	21.2 ± 27.1	4.7 ± 4.6	0.000
LDH (U/L)	200.7 ± 42.3	241.4 ± 103.2	191.1 ± 31.9	0.036
Hemoglobin (g/L)	140.3 ± 14.4	128.6 ± 14.9	120.3 ± 12.4	0.000
Albumin (g/L)	41.7 ± 3.7	38.4 ± 4.0	37.5 ± 3.8	0.005
Lymphocyte count (G/L)	1.1 ± 0.5	1.3 ± 0.4	1.4 ± 0.4	0.001
IL-6 (pg/mL)^ c^	-	8.7 ± 10.9	7.5 ± 9.4	0.457

a Median values and interquantile ranges were given for total CT scores, and mean values ± standard deviations were given for the laboratory parameters. Kruskal-Wallis rank sum test and Mann-Whitney U test were utilized for the difference. CRP = C-reactive protein; LDH = lactate dehydrogenase; IL-6 = interleukin-6. b Normal range: CRP (0-8 mg/L), LDH (109-245U/L), hemoglobin (115-250 g/L), albumin (35-55 g/L), lymphocyte count (1.1-3.2 G/L), IL-6 (0-7 pg/mL) c IL-6 was tested only in 3 patients at initial presentation.

**Table 4:** The correlation coefficients between total CT scores and the laboratory parameters at each milestone.^a^

	Total CT scores					
	Initial presentation		Worst CT manifestation		Recovery finding before discharge	
Parameters	r	p value	r	p value	r	p value
CRP	**0.58**	**0.00**	**0.47**	**0.00**	0.01	0.95
LDH	**0.64**	**0.045**	**0.65**	**0.00**	0.31	0.07
Hemoglobin	0.12	0.44	-0.15	0.12	-0.21	0.08
Albumin	0.02	0.96	**-0.43**	**0.00**	**-0.41**	**0.00**
Lymphocyte count	-0.10	0.51	-0.16	0.31	0.05	0.70
IL-6	-	-	0.27	0.09	0.36	0.07

^a^ Spearman rank correlation analysis was performed. R values with the p values ≤ 0.05 were highlighted in bold. CRP = C-reactive protein; LDH = lactate dehydrogenase; IL-6 = interleukin-6.

**Table 5:** The correlation coefficients between total CT scores, laboratory parameters and illness durations.a

Parameters	Illness duration from symptom onset to worst CT manifestation				Illness duration from symptom onset to discharge					
	Initial presentation		Worst CT manifestation		Initial presentation		Worst CT manifestation		Recovery finding before discharge	
	r	p value	r	p value	r	p value	r	p value	r	p value
CT scores	**0.33**	**0.01**	**0.29**	**0.01**	0.09	0.48	0.22	0.05	0.06	0.58
CRP	0.28	0.07	0.12	0.30	0.25	0.10	0.16	0.17	-0.13	0.34
LDH	-0.01	0.99	0.23	0.09	0.00	1.00	0.16	0.25	-0.01	0.96
Hemoglobin	0.18	0.22	**-0.34**	**0.00**	0.27	0.06	-0.03	0.82	0.05	0.68
Albumin	0.01	0.99	-0.18	0.15	-0.22	0.50	-0.19	0.13	0.18	0.20
Lymphocyte count	-0.02	0.91	0.16	0.17	-0.05	0.73	-0.11	0.34	-0.06	0.65
IL-6	-	-	0.06	0.78	-	-	-0.05	0.81	0.01	0.97

^a^ Spearman rank correlation analysis was performed. R values with the p values ≤ 0.05 were highlighted in bold. CRP = C-reactive protein; LDH = lactate dehydrogenase; IL-6 = interleukin-6.

## Abbreviations

COVID-19: coronavirus disease 2019
CRP: c-reactive protein
IL-6: interleukin-6
LDH: lactate dehydrogenase
rRT-PCR: real-time reverse transcriptase-polymerase chain reaction
SARS-CoV-2: severe acute respiratory syndrome coronavirus 2
