# Supplementary material for: Longitudinal changes in COVID-19 clinical measures and correlation with the extent of CT lung abnormalities
Source: Int J Med Sci. 2021 Jan 16;18(5):1277–84. doi: 10.7150/ijms.51279 (PMC7847610; doi:10.7150/ijms.51279)
Supplement: Supplementary file 1 — Supplementary materials, figures and tables. [file ijmsv18p1277s1.pdf]

Supplementary Table 1. Laboratory parameters reported abnormal in more than 50% of patient samples in prior studies.

Author (year)	Sample size	Laboratory parameters (percentage of deranged values)[a]
Guan (2020)	1099	Lymphocyte count (82.1%); CRP (60.7%)
Zhang (2020)	140	CRP (91.9%); Lymphocyte count (75.4%)
Chen(2020)	99	Albumin(98%); CRP (86%); LDH (76%); IL-6 (52%), Hemoglobin (51%)
Huang (2020)	41	LDH (73%); Lymphocyte count (63%)

[a] CRP = C-reactive protein; LDH = lactate dehydrogenase; IL-6 = interleukin-6

Supplementary Table 2. The required sample size by post-hoc sample size calculation with type I error rate of 0.05 and power of 0.70.

Correlation	Correlation coefficient	Sample size required
**Positive correlations between total CT scores and laboratory parameters at each milestone**		
Initial presentation		
Total CT scores vs. CRP	0.58	19
Total CT scores vs. LDH	0.64	16
Worst CT manifestation		
Total CT scores vs. CRP	0.47	29
Total CT scores vs. LDH	0.65	16
Total CT scores vs. Albumin	-0.43	36
Recovery findings before discharge		
Total CT scores vs. Albumin	-0.41	36
